# Author Correction: Enhanced NF-κB signaling in type-2 dendritic cells at baseline predicts non-response to adalimumab in psoriasis

**DOI:** 10.1038/s41467-021-27447-6

**Published:** 2021-12-16

**Authors:** Rosa Andres-Ejarque, Hira Bahadur Ale, Katarzyna Grys, Isabella Tosi, Shane Solanky, Chrysanthi Ainali, Zeynep Catak, Hemawtee Sreeneebus, Jake Saklatvala, Nick Dand, Emanuele de Rinaldis, Anna Chapman, Frank O. Nestle, Michael R. Barnes, Richard B. Warren, Nick J. Reynolds, Christopher E. M. Griffiths, Jonathan N. Barker, Catherine H. Smith, Paola Di Meglio, the PSORT Consortium, Paola Di Meglio, Richard Emsley, Andrea Evans, Katherine Payne, Deborah Stocken

**Affiliations:** 1 St. Johns Institute of Dermatology, Kings College London, London, United Kingdom; 2 NIHR Biomedical Research Centre, Guys & St Thomas NHS Foundation Trust & King’s College London, London, UK; 3 DIGNOSIS Limited, London, UK; 4 Department of Medical and Molecular Genetics, Kings College London, London, UK; 5 Dermatology Department, Queen Elizabeth Hospital, Lewisham and Greenwich NHS Trust, London, UK; 6 Centre for Translational Bioinformatics, William Harvey Research Institute, Queen Mary University of London, Charterhouse Square, London, UK; 7 Dermatology Centre, Salford Royal Hospital, The University of Manchester, Manchester Academic Health Science Centre, Manchester, UK; 8 Institute of Translational and Clinical Medicine, Newcastle University Medical School and Department of Dermatology, Royal Victoria Infirmary, Newcastle Hospitals NHS Foundation Trust, Newcastle upon Tyne, UK; 9 Department of Biostatistics and Health Informatics, Institute of Psychiatry, Psychology & Neuroscience, King’s College London, London, UK; 10 Manchester Centre for Health Economics, Division of Population Health, Health Services Research and Primary Care, The University of Manchester, Manchester, UK; 11 Leeds Institute of Clinical Trials Research, University of Leeds, Leeds, UK; 12 Present Address: Sanofi, Cambridge, MA USA

**Keywords:** Conventional dendritic cells, T cells, Predictive markers

Correction to: *Nature Communications* 10.1038/s41467-021-25066-9, published online 6 August 2021.

The original version of the Source Data file associated with this Article included an error in the ‘Fig. 6 and Supp Fig. 15 tab’, in which CD274 was inadvertently represented by a number series ranging from CD274 to CD300. The HTML has been updated to include a corrected version of Source Data; the original incorrect version of Source Data can be found as Supplementary Information associated with this Correction.

## Source data

Incorrect Source Data
Updated Source Data

