# Supplementary material for: Enhanced NF-κB signaling in type-2 dendritic cells at baseline predicts non-response to adalimumab in psoriasis
Source: Nat Commun. 2021 Aug 6;12:4741. doi: 10.1038/s41467-021-25066-9 (PMC8346545; doi:10.1038/s41467-021-25066-9)
Supplement: Supplementary file 3 — Description of Additional Supplementary Files [file 41467_2021_25066_MOESM3_ESM.pdf]

Description of Additional Supplementary Files

File Name: Supplementary Data 1
Description: Patient information and demographics
Table showing patient demographics, cohorts and which patients passed the quality control filters and were included in each step of the study.

File Name: Supplementary Data 2
Description: Effect of adalimumab on NF-kB translocation in immune cells
Table comparing NF-kB translocation (rd.score) before and after therapy for TNF and LPS stimulated immune cells. Two-tailed Wilcoxon test with FDR correction was used for the comparisons. The median Rd score for the 2 visits being compared as well as the percentage of inhibition are also shown. Related to Figure 1b,c.

File Name: Supplementary Data 3
Description: Correlation analysis NF-kB translocation
Table showing the results of the linear regression analysis of NF-kB translocation (rd.score) before and after treatment and response to adalimumab at week 12 in immune cells. FDR was used to correct for multiple testing. Related to Figure 1d.

File Name: Supplementary Data 4
Description: Covariate analysis NFkB translocation and drug levels.
Table showing the results of the covariate linear regression analysis showing the correlation of drug levels (DL) and NF-kB translocation (rd.score) before and after treatment and response to adalimumab at week 12 in immune cells. FDR was used to correct for multiple testing.

File Name: Supplementary Data 5
Description: Effect of adalimumab on NFkB phosphorylation
Table comparing NF-kB phosphorylation before and after therapy for TNF and LPS stimulated immune cells. Two-tailed Wilcoxon test with FDR correction was used for the comparisons. The median log10 fold change for the 2 visits being compared is shown. Related to Supplementary Figure 5.

File Name: Supplementary Data 6
Description: Correlation analysis NF-kB phosphorylation
Table showing the results of the linear regression analysis of NF-kB phosphorylation (log10FC_UNS) before and after treatment and response to adalimumab at week 12 in immune cells. FDR was used to correct for multiple testing. Related to Figure 2.

File Name: Supplementary Data 7
Description: Covariate analysis - correlation NF-kB phosphorylation
Table showing the results of the covariate linear regression analysis showing the correlation of HLA-Cw6 status (HLA_C_0602), presence of concomitant psoriatic arthritis (psoriatic.arthritisYes), disease severity before therapy (PASI.baseline), previous therapy (biologic.naiveYes), smoking (smokerYes), age, ethnicity (ethnicityWhite), weight, gender (genderM) and NF-kB translocation (log10FC_UNS) before and after treatment and response to adalimumab at week 12 in immune cells. FDR was used to correct for multiple testing.

File Name: Supplementary Data 8
Description: Correlation analysis T cell subsets
Table showing the results of the linear regression analysis of frequency of IL17 and IL10 producing T cell subsets before and after treatment and response to adalimumab at week 12 in immune cells. FDR was used to correct for multiple testing. Related to Figure 4.

File Name: Supplementary Data 9
Description: Effect of adalimumab on dendritic cell phenotype

Table comparing DC maturation marker expression before and after therapy. Two-tailed Wilcoxon test with FDR correction was used for the comparisons. The median expression of each marker for the 2 visits being compared as well as the percentage of inhibition are also shown. Related Supplementary Figure 12.

File Name: Supplementary Data 10
Description: Correlation analysis DC phenotype
Table showing the results of the linear regression analysis of DC maturation marker expression before and after treatment and response to adalimumab at week 12. FDR was used to correct for multiple testing. Related to Figure 4.

File Name: Supplementary Data 11
Description: Dendritic cell phenotype in PASI75 responders and no responders
Table comparing DC maturation marker expression before and after therapy in adalimumab PASI75 responders and non-responders (week 12). Two-tailed Wilcoxon test with FDR correction was used for the comparisons. The median expression of each marker for the 2 groups being compared is also shown. Related to Figure 4.

File Name: Supplementary Data 12
Description: Antibodies used in the study
Table showing the details of all the antibodies used in the study, including manufacturer, clone, catalogue number and concentration used.
