# Supplementary material for: Enhanced NF-κB signaling in type-2 dendritic cells at baseline predicts non-response to adalimumab in psoriasis
Source: Nat Commun. 2021 Aug 6;12:4741. doi: 10.1038/s41467-021-25066-9 (PMC8346545; doi:10.1038/s41467-021-25066-9)
Supplement: Supplementary file 16 — Reporting Summary [file 41467_2021_25066_MOESM16_ESM.pdf]

nature research

Corresponding author(s): Paola Di Meglio

Last updated by author(s): May 11, 2021

Reporting Summary

Nature Research wishes to improve the reproducibility of the work that we publish. This form provides structure for consistency and transparency in reporting. For further information on Nature Research policies, see our Editorial Policies and the Editorial Policy Checklist.

Statistics

For all statistical analyses, confirm that the following items are present in the figure legend, table legend, main text, or Methods section.

n/a	Confirmed	
☐	☒	The exact sample size (*n*) for each experimental group/condition, given as a discrete number and unit of measurement
☐	☒	A statement on whether measurements were taken from distinct samples or whether the same sample was measured repeatedly
☐	☒	The statistical test(s) used AND whether they are one- or two-sided<br>*Only common tests should be described solely by name; describe more complex techniques in the Methods section.*
☐	☒	A description of all covariates tested
☐	☒	A description of any assumptions or corrections, such as tests of normality and adjustment for multiple comparisons
☐	☒	A full description of the statistical parameters including central tendency (e.g. means) or other basic estimates (e.g. regression coefficient) AND variation (e.g. standard deviation) or associated estimates of uncertainty (e.g. confidence intervals)
☐	☒	For null hypothesis testing, the test statistic (e.g. *F*, *t*, *r*) with confidence intervals, effect sizes, degrees of freedom and *P* value noted<br>*Give P values as exact values whenever suitable.*
☐	☒	For Bayesian analysis, information on the choice of priors and Markov chain Monte Carlo settings
☐	☒	For hierarchical and complex designs, identification of the appropriate level for tests and full reporting of outcomes
☐	☒	Estimates of effect sizes (e.g. Cohen's *d*, Pearson's *r*), indicating how they were calculated

Our web collection on statistics for biologists contains articles on many of the points above.

Software and code

Policy information about availability of computer code

Data collection	Imaging flow cytometry data were collected using Amnis INSPIRE software 4.0, flow cytometry data were collected using BD FACS Diva 8.0.1, Immunofluorescence data were acquired using NIS Elements Ver4.60.00. No custom software was used.
Data analysis	Imaging flow cytometry data were analysed using Amnis IDEAS software 4.0, flow cytometry data were analysed using Flowjo™ software 10.6.1, Immunofluorescence data were analysed using NIS Elements Ver4.60.00. Genotype imputaion was performed using Michigan Imputation Server . All other analyses were done in R 3.6.0. R packages used: flowAI 1.20.1, flowCore 2.2.0, limma 3.46.0, FlowSOM 1.22.0, ConsensusClusterPlus 1.54.0, pROC R 1.17.0.1. No custom software was used.

For manuscripts utilizing custom algorithms or software that are central to the research but not yet described in published literature, software must be made available to editors and reviewers. We strongly encourage code deposition in a community repository (e.g. GitHub). See the Nature Research guidelines for submitting code & software for further information.

Data

Policy information about availability of data

All manuscripts must include a data availability statement. This statement should provide the following information, where applicable:

- Accession codes, unique identifiers, or web links for publicly available datasets
- A list of figures that have associated raw data
- A description of any restrictions on data availability

Source data are provided with this paper. Whole blood eQTL summary statistics for CD274 gene are available at www.gtexportal.org. All other data are provided in the article and its Supplementary files. Summary statistics for CD274 genotype in the PSORT genetic dataset and raw .fcs files can be obtained from the corresponding author upon reasonable request.

Field-specific reporting

Please select the one below that is the best fit for your research. If you are not sure, read the appropriate sections before making your selection.

☒ Life sciences ☐ Behavioural & social sciences ☐ Ecological, evolutionary & environmental sciences

For a reference copy of the document with all sections, see nature.com/documents/nr-reporting-summary-flat.pdf

Life sciences study design

All studies must disclose on these points even when the disclosure is negative.

Sample size	No statistical methods were used to calculate sample size, which was instead based on availability of sample within the recruited cohort and on previous experiments performed on human samples with comparable expected effect size (Di Meglio et al 2013).
Data exclusions	As stated in the manuscript, for imaging flow cytometry experiments, populations with fewer than 10 cells were excluded from the analysis as they produced higher median associated errors providing unreliable median internalization score values. For flow cytometry experiments, samples with viability < 85% were considered apoptotic and excluded from the analysis.
Replication	For each experimental read-out, each patient samples has been assayed once, alongside internal controls run in each batch to control for inter-experiment variability, which resulted to be minimal, confirming reproducibility of the essays. Replication of the biomarker has been achieved with the acquisition of independent replication and clinical validation cohorts that confirmed findings obtained in the discovery cohort.
Randomization	Samples were equally randomized among different runs.
Blinding	All experiments and initial data pre-processing and analyses were performed blinded with regards to response to treatment and disease status. Unblinding was required for the further statistical analysis. However, the automated-data analysis workflow employed strongly reduces the potential investigator bias.

Reporting for specific materials, systems and methods

We require information from authors about some types of materials, experimental systems and methods used in many studies. Here, indicate whether each material, system or method listed is relevant to your study. If you are not sure if a list item applies to your research, read the appropriate section before selecting a response.

Materials & experimental systems

n/a	Involved in the study	
☐	☒	Antibodies
☒	☐	Eukaryotic cell lines
☒	☐	Palaeontology and archaeology
☒	☐	Animals and other organisms
☐	☒	Human research participants
☒	☐	Clinical data
☒	☐	Dual use research of concern

Methods

n/a	Involved in the study	
☒	☐	ChIP-seq
☐	☒	Flow cytometry
☒	☐	MRI-based neuroimaging

Antibodies

Antibodies used	The information for all the antibodies used in the study can be found in supplementary data 12
Validation	Commercial antibodies were validated by the manufactures as indicated on their web sites. BD biosciences and Biolegend validate antibodies by testing on a combination of primary cells, cell lines and/or transfectant cell models with relevant controls using multiple immunoassays to ensure biological accuracy. They also perform multiplexing with additional antibodies to interrogate antibody staining in multiple cell populations. D14E12 antibody from CellSignalling was validated by the manufacturer by Flow cytometric analysis of HeLa cells compared to concentration matched Rabbit (DA1E) mAb IgG XP® Isotype Control #3900. LS-A9382 and LS B7397 from LSBio were validated by the manufacturer by IHC of formalin-fixed, paraffin-embedded human prostate and human t lymphocytes, respectively, after heat-induced antigen retrieval. ab45420 and ab205921 from Abcam were validated by the manufacturer by immunofluorescence staining of mouse bone marrow derived macrophages and PD-L1 stably expressed Chinese hamster ovary epithelial cell labelling PD-L1, respectively, using Goat anti rabbit IgG (ab150077) as secondary antibody and PBS instead of the primary antibody as negative control. 14-0116-82 from eBioscience was validated by the manufacturer using flow cytomtetric analysis of normal human peripheral blood cells. Appropriate isotype controls were used. A-21127 and A-21245 from Thermofisher were validated by the manufacturer by immunofluorescence staining of HeLa stained with alpha Tubulin (236-10501) Mouse Monoclonal Antibody. In addition, all antibodies were titrated in-house before use in the experimental panels

Human research participants

Policy information about studies involving human research participants

Population characteristics	Clinical data from all participans can be found in supplementary data 1
Recruitment	Patients with chronic plaque type psoriasis with moderate-severe disease (PASI>10) were recruited into the Psoriasis Stratification to Optimise Relevant Therapy (PSORT) study at 6 centers in the UK between May 2015 and July 2018 and due to start biologic therapy (ustekinumab or adalimumab) as part of routine clinical practice. Exclusion criteria were the use of systemic or biological therapy for psoriasis for 2 weeks prior to study entry, use of PUVA therapy for 3 months or UV-B for 1 month prior to study entry or use of topical treatments to site of biopsies (except for emollients) for 2 weeks prior to study entry, as well as serious/uncontrolled systemic disease or medical condition. Healthy volunteers were were recruited as age, ethnicity and gender-matched controls to the PSORT adalimumab combined cohort. No self-selection bias were present in recruiting psoriasis patients and healthy volunteers.
Ethics oversight	This study was approved by the London Bridge research ethics committee (REC numbers: 14/LO/1685; 11/LO/1692; 06/Q0704/18; 13/EE/0241).

Note that full information on the approval of the study protocol must also be provided in the manuscript.

Flow Cytometry

Plots

Confirm that:

- [x] The axis labels state the marker and fluorochrome used (e.g. CD4-FITC).
- [x] The axis scales are clearly visible. Include numbers along axes only for bottom left plot of group (a 'group' is an analysis of identical markers).
- [x] All plots are contour plots with outliers or pseudocolor plots.
- [x] A numerical value for number of cells or percentage (with statistics) is provided.

Methodology

Sample preparation	Blood was collected at participating sites in BD Vacutainer™ Hemogard Closure Plastic K2-Edta Tubes (BD Biosciences, CA). For the discovery cohort, an aliquot of fresh blood was used for imaging flow cytometry (see below). Peripheral blood mononuclear cells (PBMCs) were isolated within 4 hours of collection using Ficoll-Paque density gradient centrifugation in Leucosep tubes (Greiner Bio-One, Austria). Cells were viably cryopreserved as previously described 44 and stored in liquid nitrogen until used or shipped to St John's Institute of Dermatology. Prior to each experiment, cell count and viability were measured with Via1-Cassette™ on a NucleoCounter NC-200 (Chemometec, DK).
Instrument	BD LSRFortessa and BD Canto
Software	BD FACS Diva 8.0.1, Flowjo™ software 10.6.1 and R 3.6.0
Cell population abundance	For imaging flow cytometry experiments, 200 ul of fresh whole blood were used. For flow cytometry experiments, 3 million cells were aliquoted per condition. For the generation and maturation of monocyte-derived dendritic cell, 250.000 CD14+ monocytes per condition were seeded in a 24-well plate.
Gating strategy	Representative gating strategy and cell hierarchy for each of the panel is shown in Supplemental Figure 2, 4, 9, 18b and 21b. In brief, for imaging flow cytometry experiments, cells in focus were gated using the gradient RMS of the DAPI channel, then singlets using the aspect ratio and the area and then cells which were double positive for DAPI and the nuclear staining NF-kB. Cell types of interest were gated based on the expression of canonical immune markers. For flow cytometry experiments, including phosphor flow cytometry, cells were gated using FSC-A and SSC-A, then singlets using SSC-A and SSC-W and then live cells using Live/Dead FVS780 marker. Then, cell types of interest were gated based on the expression of canonical immune markers. A Live/Dead marker was not included in the phopho flow cytometry panel as it was not compatible with the sort stimulation times. Boundaries between positive and negative cells for all markers were stablished using FMO controls in the same sample type.

- [x] Tick this box to confirm that a figure exemplifying the gating strategy is provided in the Supplementary Information.
